# Effectiveness of NexQuest Natural Frequency Technology^®^ on sleep and mood of adults with insomnia symptoms: a randomized, double blind and placebo controlled crossover trial

**DOI:** 10.5935/1984-0063.20190125

**Published:** 2020

**Authors:** Stephanie Hooper, Tarah Lynch, Kevin Coyle, David Hooper, Heather A Hausenblas

**Affiliations:** Jacksonville University, Applied Health Sciences - Jacksonville - FL - United States.

**Keywords:** Sleep Quality, Natural Frequency Technology

## Abstract

**Objectives:**

The study purpose was to conduct a four-week randomized double-blind placebo-controlled crossover trial on adults with insomnia symptoms to examine the effectiveness of Natural Frequency Technology^(®)^ (NFT), found in Philip Stein Sleep Bracelets, on sleep quality, anxiety/stress levels, and mood.

**Methods:**

Adults (*N* = 44, *M* age = 41.9 years) were randomized to the Placebo Bracelet (PB) or NFT Sleep Bracelet (SB) for two weeks and then the alternative bracelet for two weeks. Self-reported mood, anxiety/stress, and sleep quality were completed at Day 0 (PRE) and following each condition; POST PB and POST SB).

**Results:**

When the participants wore the SB, compared to the PB, they had improved sleep quality (i.e., Pittsburgh Sleep Quality Index), anxiety/perceived stress, and mood, *p*’s < .05.

**Discussion:**

The SB may be simple, noninvasive, and non-pharmacological intervention to improve sleep quality and daytime mood.

## INTRODUCTION

Sleep deprivation is associated with increased risk in all-cause mortality, worse quality of life and decreases in socioeconomic consequences^1^^-^3. Common interventions to improve sleep include over-the-counter and prescribed drugs, which are criticized for their side effects, short- and long-term efficacy, and dependency.

A drug-free approach to healthcare and sleep wellness may be electromagnetic (EM) field therapy. With the discovery that the human EEG shares similar fundamental EM frequencies with earth’s natural EM field^4^, researchers have found this relationship provides therapeutic benefits, including improved heart rate variability and lower blood pressure^5^^,^^6^. These natural, low-frequency EM fields have been reproduced and used clinically, as seen with the success of transcranial magnetic stimulation. The EM fields produced via transcranial magnetic stimulation increase slow wave activity during sleep, an important player in the body’s restorative process^7^. With EM field therapy, the nervous system resonates with external EM frequencies, amplifying and propagating their signals throughout the body^8^. Since the frequencies are strengthened through the body’s coordinating network of neurons, weak EM fields, even as low as 1µT, are able to produce significant therapeutic benefits^9^^,^^10^. Further exploring the effect of weak EM fields, two recent studies found positive health effects of EM therapy when applied through a charged medium^11^^,^^12^. More specifically, by using areas of earth’s natural EM fields to enhance mattresses with subtle EM frequencies, the researchers found that participants had less anxiety and fell asleep more easily.

The Natural Frequency Technology^®^(NFT) found in Philip Stein^TM^ Bracelets may provide a healthier and natural alternative to over-the-counter and prescription sleep aids with the use of subtle natural EM frequencies. The Philip Stein^TM^ Sleep Bracelet is embedded with unique Natural Frequency Technology®, which resonates EM frequencies to the body to promote improved sleep quality. The Sleep Bracelet resembles a wristwatch, but the face is a metal disc that contains subtle natural EM fields which resonate with the nervous system, amplifying its signal. For consistency capitalize Sleep Bracelet is programmed with specific natural frequencies that may help the body regulate sleep and wake cycles, thus inducing deep, restful and uninterrupted sleep^13^. A laboratory study by Breus and Rubik^14^ revealed that participants who wore the original Philip Stein Sleep Bracelet reported improvements on a variety of sleep parameters, albeit these findings were nonsignificant.

The study purpose was to conduct a four-week randomized double-blind placebo-controlled crossover trial on adults with insomnia symptoms to examine the effectiveness of NFT designed and manufactured by NexQuest Life Sciences (163 Burlington Path Road Cream Ridge, NJ 08514, USA, https://www.nexquest.com) on sleep quality, anxiety/stress levels and mood. We hypothesized that when adults wore the NFT Sleep Bracelet (SB) it would result in improved sleep quality and mood compared to when participants wore the Placebo Bracelet (PB).

## METHODS

### Participants

Participants were 44 adults (*n* = 24 women and *n* = 20 men, *M* age = 41.9 years) who reported insomnia symptoms (i.e., scored ≥ 7 on the Insomnia Severity Index). Individuals were excluded if they smoked, had sleep or health disorders, were at high risk for sleep apnea (as determined by the Berlin Sleep Apnea Questionnaire^15^), had a BMI greater than 32, or were good sleepers (as determined by the Insomnia Severity Index^16^). See Figure 1 for the Participant Flow Chart. The participants Insomnia Severity Index scores reflected moderate insomnia at baseline, with a mean score of 12.34 (*SD* = 4.15). Regarding insomnia issues, 80% had difficulties falling asleep, 91% had difficulty staying asleep and 87% woke up too early.

**Figure:**
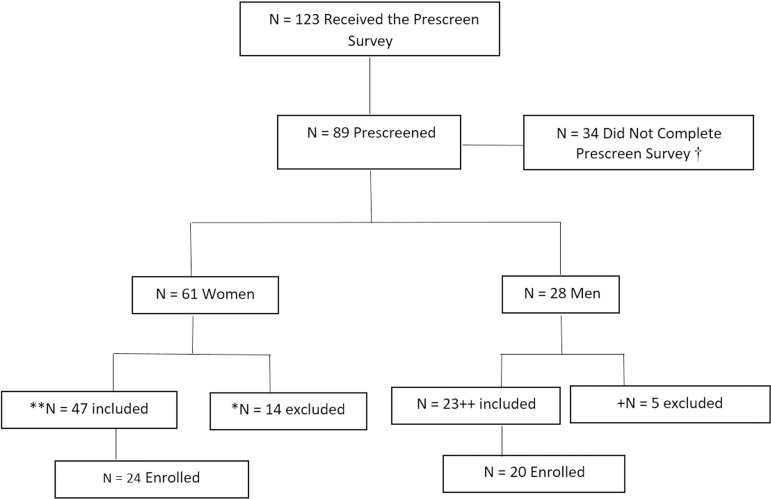
Note: † = 16 women and 18 men did not complete the prescreening survey * N = 14 excluded because they did not meet the inclusion criteria (N = 8 excluded due to high BMI, N = 2 excluded due to high BMI and sleep apnea, N = 4 excluded for scoring too low on insomnia index) ** N = 3 excluded (N = 2 excluded for not responding after the prescreening survey, 1 was out of town during Day 0 Assessments) + N = 5 excluded because they did not meet the inclusion criteria (N = 1 excluded due to high BMI and sleep apnea, N = 4 excluded for scoring too low on the insomnia index) ++ N = 3 excluded (N = 2 excluded for not scheduling Day 0 assessments, N = 1 excluded due to international travel during the study period)

### Procedures

Institutional Review Board (IRB) approval to conduct this study was obtained and all the participants signed the IRB approved consent form prior to study participation. Using a placebo-controlled crossover design, participants were randomized to either the PB or SB condition for two weeks. Following a two day “washout period” the participants wore the alternative bracelet for two weeks. The bracelets were identical except that the SB contained the NFT that is not recognizable. Both the participants and the research team were blinded to the conditions.

Participants wore the bracelet immediately before their nighttime sleep. Upon awakening they removed the bracelet. They received a daily text reminder in the evening to wear their bracelet immediately before their nighttime sleep. And they also received a daily morning text with a link to complete the daily diary survey that assessed their adherence to wearing the sleep bracelet and any issues with wearing the bracelet the prior night. They also completed standardized self-report assessments of their mood, anxiety/stress, and sleep quality at baseline (PRE) and following each of the conditions (POST SB and POST PB). Participants maintained their current lifestyle behaviors for the study duration.

### Measures

*Daily Diary*: Each morning the participants completed the Daily Diary that assessed their adherence to wearing the sleep bracelet (Did you wear the sleep bracelet last night?) and any issues with wearing the bracelet. If a participant indicated “Yes” to having an issue with the sleep bracelet they were then prompted with an open ended response to describe the issue.

The following psychometrically validated self-report measures were completed at Day 0 (PRE) and following each condition (i.e., POST SB and POST PB):

*Pittsburgh Sleep Quality Index (PSQI):* PSQI is a widely used inventory to assess changes in subjective sleep quality in both clinical practice and research. It is a self-report questionnaire with 19 items (scale range = 0 - 21). Higher scores indicate more sleep problems, and a score > 5 separates poor sleepers from good sleepers^17^. If participants scored “5” or more it is suggested that they discuss their sleep habits with a healthcare provider. The PSQI has good psychometric properties and it is recommended as an essential outcome measures in sleep studies^17^.

*Profile of Mood States (POMS):* The POMS is a well-established measure of psychological distress derived from factor analysis, and its high levels of reliability and validity have been documented^18^. This questionnaire contains 65 words/adjectives that describe several aspects of mood that are grouped into the following six subscales: tension, depression, anger, vigor, fatigue and confusion. The vigor subscale refers to a positive state of mind, and the other factors, to a negative state of mind. Each item is valued following a Likert type format, with five response alternatives: not at all (0), a little (1), moderately (2), quite a bit (3) or extremely (4). The subscales are combined for a total mood score.

*Perceived Stress Scale*: The Perceived Stress Scale (PSS) is the most widely used validate psychological instrument for measuring the perception of stress^20^. It is a measure of the degree to which situations in one’s life are appraised as stressful. Items were designed to tap how unpredictable, uncontrollable and overloaded respondents find their lives. The scale also includes a number of direct queries about current levels of experienced stress.

*Trait Anxiety Inventory*: The Trait Anxiety Inventory is widely used to measure anxiety symptoms^19^. The Trait Anxiety Inventory contains 20 four-point Likert scale items. Trait anxiety items assess how subjects generally feel. Participants were asked to indicate their level of anxiety over the last 2 weeks. Total scores range from 20 to 80. Higher scores indicate more severe anxiety levels.

### Data Analysis

Data were analyzed for normality using Shapiro-Wilk Test and the skewness and kurtosis values of the scales. Descriptive statistics were expressed in Mean (*SD*) format. The data were normally distributed thus paired *t*-tests were used to analyze mean differences between groups. The self-report measures consisted of Likert-scale items which produce quantitative values based on participant responses. Each survey was totaled individually according to the questionnaire specifications, and change scores for each participant were determined by comparing baseline responses (PRE) to those reported following each intervention condition (i.e., POST SB and POST BB). These change scores were then compared using SPSS (Version 24) to determine condition differences via paired *t*-tests (*p*’s ≤ .05). No data were missing from the self-report data, as there was 100% adherence rates for all surveys.

Power analyses indicated a sample size of 34 was needed to achieve a power of 80% and a level of significance of 5% (two sided), for detecting an effect size of 0.5 between pairs for a t-distribution^18^. To control for potential dropout 44 participants were enrolled in the study.

## RESULTS

The nightly adherence rate to wearing the SB was 93.9% and for the PB was 91.1%. When the participants wore the SB, compared to the PB, they had significant improvements from baseline (PRE) to POST condition in their sleep quality (*t*(43) = 2.15, *p* = .03), anxiety (*t*(43) = 2.19, *p* = .03), perceived stress (*t*(43) = 2.68, *p* = .01) and mood (*t*(43) = 2.19, *p* = .03, see Table 1). At baseline 84% (*n* = 37) of the participants had poor sleep quality (as determined by the PSQI). Significantly less participants were classified as having poor sleep quality after wearing the SB (*n* = 21) compared to the PB (n = 31), with a Pearson Chi square analysis value of *X*^2^= 0.03. No issues were reported when the participants wore the bracelet.

**Table 1:** Descriptive statistics for the anxiety, perceived stress, mood and sleep quality outcomes.

Outcome	Change Score Sleep Bracelet	Change Score Placebo Bracelet
	Mean (*SD*)	Mean (*SD*)
Anxiety*	-5.52 ± 1.86 (6.11)	-3.80 ± 1.80 (5.94)
Perceived Stress*	-3.41 ± 1.72 (5.66)	-1.64 ± 1.45 (4.78)
Profile of Mood States*	-15.34 ± 5.71 (18.77)	-9.36 ± 4.31 (14.18)
Pittsburg Sleep Quality Index*	-3.00 ± 1.00 (3.30)	-1.86 ± 0.86 (2.83)

* Significant difference between SB and PB change scores; *SD* = Standard deviation.

## DISCUSSION

Consistent with the hypothesis, the NFT bracelet resulted in improved sleep quality, mood, anxiety and perceived stress in otherwise healthy adults who reported experiencing insomnia symptoms. These findings are consistent with research showing relationships between poor sleep and depression and negative mood^19^. These significant but small improvements in sleep quality are similar to those found in meta-analyses examining complementary and alternative medicines such as melatonin, valerian, yoga and meditation^20^^,^^21^.

Strengths of the study include a randomized cross-over design in the home environment. A main limitation is the lack of an objective sleep measure. Future researcher should examine how NFT longitudinally impacts the physiology and pathways associated with sleep in a variety of populations and environments (e.g., hospitals) using both objective and self-report measures. In summary, the SB was well-tolerated and is a simple, noninvasive, and non-pharmacological intervention to promote improved sleep quality/quantity, mood, anxiety and stress with adults who experience insomnia symptoms.
